# P-NGAL Day 1 predicts early but not one year graft function following deceased donor kidney transplantation – The CONTEXT study

**DOI:** 10.1371/journal.pone.0212676

**Published:** 2019-02-28

**Authors:** Marie B. Nielsen, Nicoline V. Krogstrup, Gertrude J. Nieuwenhuijs-Moeke, Mihai Oltean, Frank J. M. F. Dor, Bente Jespersen, Henrik Birn

**Affiliations:** 1 Department of Renal Medicine, Aarhus University Hospital, Aarhus, Denmark; 2 Department of Clinical Medicine, Aarhus University, Aarhus, Denmark; 3 Department of Anaesthesiology, University of Groningen, University Medical Center Groningen, Groningen, The Netherlands; 4 The Transplant Institute, Sahlgrenska University Hospital, Gothenburg, Sweden; 5 Division of HPB & Transplant Surgery, Department of Surgery, Erasmus MC, University Medical Center, Rotterdam, The Netherlands; 6 Imperial College Renal and Transplant Centre, Hammersmith Hospital, Imperial College, London, United Kingdom; 7 Department of Biomedicine, Aarhus University, Aarhus, Denmark; Medizinische Universitat Graz, AUSTRIA

## Abstract

**Background:**

Early markers to predict delayed kidney graft function (DGF) may support clinical management. We studied the ability of four biomarkers (neutrophil gelatinase associated lipocalin (NGAL), liver-type fatty acid-binding protein (L-FABP), cystatin C, and YKL-40) to predict DGF after deceased donor transplantation, and their association with early graft function and GFR at three and twelve months.

**Methods:**

225 deceased donor kidney transplant recipients were included. Biomarkers were measured using automated assays or ELISA. We calculated their ability to predict the need for dialysis post-transplant and correlated with the estimated time to a 50% reduction in plasma creatinine (tCr50), measured glomerular filtration rate (mGFR) and estimated GFR (eGFR).

**Results:**

All biomarkers measured at Day 1, except urinary L-FABP, significantly correlated with tCr50 and mGFR at Day 5. Plasma NGAL at Day 1 and a timed urine output predicted DGF (AUC = 0.91 and AUC 0.98). Nil or only weak correlations were identified between early biomarker levels and mGFR or eGFR at three or twelve months.

**Conclusion:**

High plasma NGAL at Day 1 predicts DGF and is associated with initial graft function, but may not prove better than P-creatinine or a timed urine output. Early biomarker levels do not correlate with one-year graft function.

**Trial registration:**

ClinicalTrials.gov NCT01395719

## Introduction

Delayed graft function (DGF) is a frequent complication after deceased donor kidney transplantation. Incidence ranges from 28–38% in kidneys from brain dead donors (DBD)[1–3], and up to 85% in kidneys from donors after circulatory death (DCD)[4–6]. DGF is related to ischemia-reperfusion injury[7–9] and is associated with prolonged hospitalization in addition to an increased risk of complications and acute rejection[7,10–12]. Moreover, in some studies DGF is associated with reduced long-term graft function and graft survival[13].

DGF is most frequently defined as “the need for dialysis during the first week after transplantation”. The time to a 50% reduction in plasma (P-) creatinine (tCr50) has been proposed as an additional definition correlating with one year graft function [14]. Unfortunately, DGF defined by these criteria cannot be assessed until several days after transplantation[15,16]. Furthermore, changes in P-creatinine during the early post-transplant period do not always correspond to changes in glomerular filtration rate (GFR), and may represent pre-renal and quickly reversible changes, as well as kidney cellular damage[16,17]. Early prediction of DGF may help to optimise clinical management immediately after transplantation and will allow early preparation for dialysis.

Several, renal biomarkers have been associated with ischemia-reperfusion injury in kidney transplantation, but their ability to predict DGF has not been well established[16,18,19]. P-neutrophil gelatinase associated lipocalin (NGAL) levels are elevated in patients with end stage renal disease[20]. High concentrations of NGAL in serum and urine on the first post-transplant day have been associated with risk of DGF[3,21–24]. Increased urinary (U) liver-type fatty acid-binding protein (L-FABP) levels have been identified in renal transplant patients with low graft function[25] and are associated with increased ischemia time, reduced peritubular capillary blood flow, and longer hospitalization in renal transplant recipients[26]. U-cystatin C excretion predicted the need for renal replacement therapy in patients with acute tubular necrosis[27]. Increased U-chitinase-3-like protein 1 (YKL-40) concentrations have been observed in the first 24 hours post-transplant in patients with DGF when compared to patients with slow or immediate graft function[28].

Our aim was to evaluate the levels and changes in 1) U- and P-NGAL, 2) U-L-FABP, 3) U-cystatin C, and 4) U-YKL-40 following deceased donor kidney transplantation and to correlate these biomarkers with DGF, early graft function, including measured GFR and estimated GFR after one year. Furthermore, we compared these biomarkers to established clinical markers such as post-transplant P-creatinine, urine output, and U-albumin/creatinine ratio.

## Materials and methods

### Study design

This study analyzed samples and outcome measurements from patients included in the CONTEXT trial (ClinicalTrials.gov: NCT01395719)[29]. This European multicenter randomized controlled trial studied the effect of remote ischemic conditioning by repetitive inflation and deflation of a cuff around the thigh of the recipient. The intervention was without any effect on the primary endpoint of tCr50 or other markers of early graft function including DGF[29]. The CONTEXT study was approved by the relevant national data protection agencies and ethical committees in the countries involved (Denmark: The National Committee on Health Research Ethics; Sweden: Regional Ethical Board; the Netherlands: METCUMCG). Informed and written consent was obtained prior to inclusion and the study was conducted in adherence with the Declaration of Helsinki.

### Inclusion

Patients undergoing deceased donor kidney transplantation were included from June 12, 2011, to December 28, 2014, at four transplant centres: Aarhus, Denmark; Gothenburg, Sweden; and Groningen and Rotterdam, the Netherlands[29].

Demographic data, P-creatinine levels and information on any dialysis procedures, were collected from hospital records. Donor characteristics were obtained from ScandiaTransplant (Aarhus and Gothenburg) and donor forms from Eurotransplant (the Netherlands). Kidney graft function was estimated at three and twelve months (to January 31, 2016) using P-creatinine, mGFR, and eGFR.

### Blood and urine sampling

Plasma and urine samples for biomarker evaluation were collected at four time points: after induction of anesthesia and insertion of a urinary catheter prior to transplantation (baseline), 90 minutes after reperfusion of the kidney and Day 1 and Day 3 after transplantation (S1 Fig, S1 Table). Samples were stored at room temperature for a maximum of one hour, centrifuged at 2800G at 4°C for ten minutes, and stored at -80°C.

P-creatinine was measured once or twice daily during the first week after transplantation, twice weekly until 30 days after transplantation, or in the case of dialysis after transplantation, until 30 days after the last dialysis[29].

A 24hr urine sample was collected on Day 1 from patients included in Aarhus and Gothenburg (S1 Table). Urine output was calculated as the average milliliter output per hour during the collection period.

### Delayed graft function

DGF was defined as the need for dialysis within the first week after transplantation.

### Biochemical analyses

NGAL was measured in plasma and urine at the Department of Clinical Biochemistry, Aarhus University Hospital (AUH) using a particle-enhanced turbidimetric immunoassay (BioPorto Diagnostics A/S, Hellerup, Denmark). U-cystatin C was measured at the Department of Clinical Biochemistry at Viborg Regional Hospital using an automated particle-enhanced turbidimetric immunoassay (Gentian, Moss, Norway). U-YKL-40 was measured using a commercial sandwich ELISA (Bio-Techne, Minneapolis, USA). The YKL-40 kit was validated for measurements in urine before analysing the samples. The inter- and intra-assay coefficients of variance (CV%) were estimated to ≤7.1% and ≤8.2% respectively. U-L-FABP was measured using sandwich ELISA (CMIC HOLDINDS Co., Ltd., Tokyo, Japan) with an inter- and intraassay CV% of ≤12.7% and ≤10.3%, respectively. All analyses were performed according to manufacturer’s instructions.

P-creatinine, U-creatinine, and U-albumin were measured at the local Department of Clinical Biochemistry using automated, standard clinical assays.

All urinary biomarkers were normalized to U-creatinine level. tCr50 was calculated by modelling the changes in P-creatinine for each patient as previously described[29].

### Glomerular filtration rate

GFR was measured after transplantation in patients with definite evidence of kidney graft function using ^51^Chrome-ethylenediamine tetraacetic acid (^51^Cr-EDTA) plasma clearance[30]. The mGFR was standardized to body surface area.

eGFR was calculated for all patients using the Modification of Diet in Renal Disease (MDRD) formula[31] without correction for race (>90% of included patients were Caucasian).

### Statistical analyses

Donor and recipient characteristics are presented as n (%), mean (SD), or median (interquartile range). Data, which were not normally distributed were transformed by logarithmic or square root transformation. Continuous variables were correlated using simple regression, while multiple linear regression was applied to adjust for confounders or predictors and to combine different biomarkers Linearity and distribution of the residuals was tested. We compared biomarker levels between two groups using Student’s t-test and evaluated the ability of the biomarkers to predict DGF using ROC analysis. The optimal cut-off was defined as the largest sum of sensitivity and specificity. Data analyses were performed using Stata version 13 software for Windows (StataCorp LP).

## Results

### Recipient and donor characteristics

**Fig 1:**
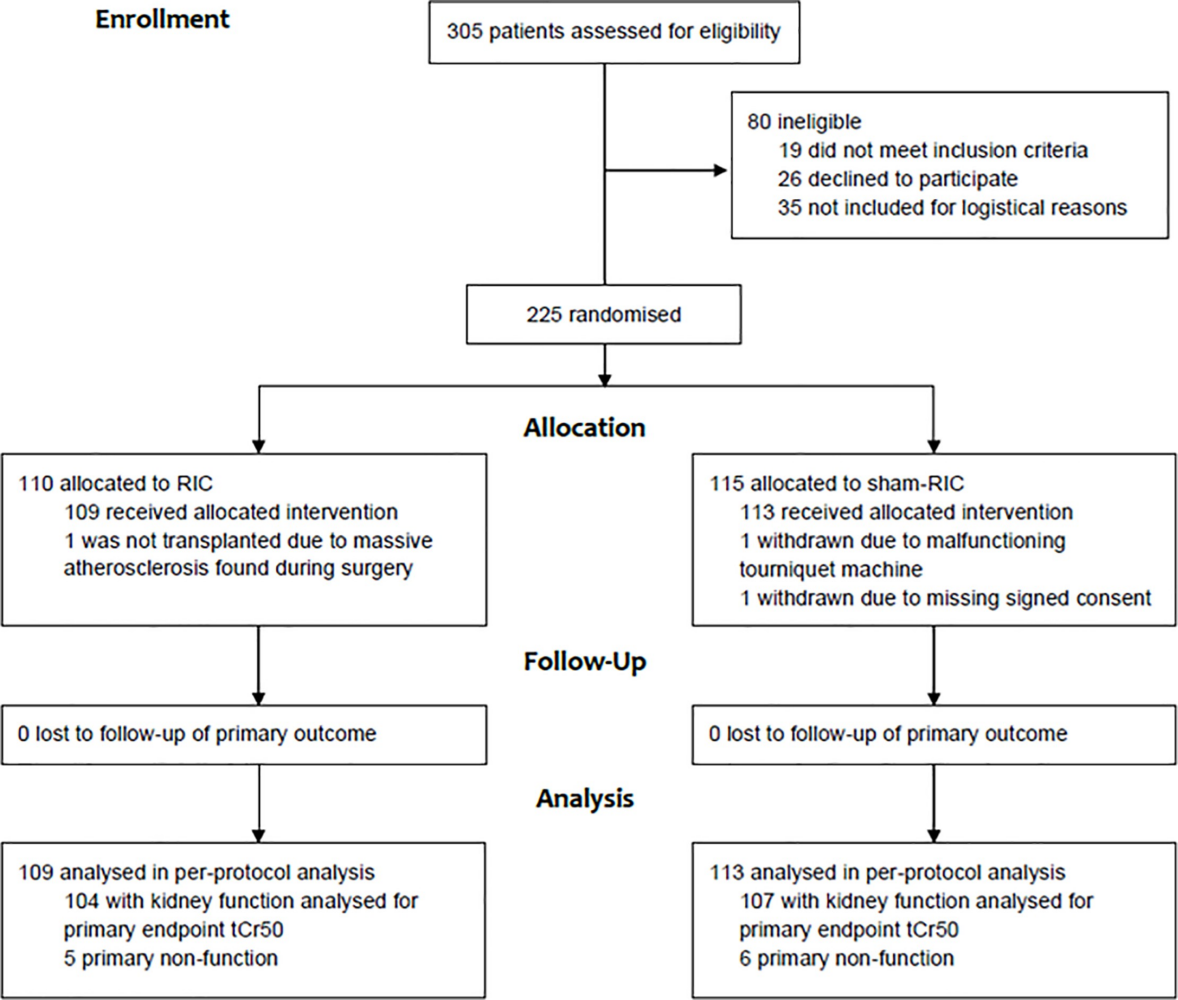
Flowchart of inclusion and randomization.

A total of 225 recipients were included in the CONTEXT trial, hereof only three was withdrawn from the study (Fig 1). 200 received a kidney from a DBD and 22 received a kidney from a DCD donor. Donor and recipient characteristics are listed in Table 1. Eleven patients (nine DBD kidney recipients and two DCD kidney recipients) were excluded from the analyses of tCr50 due to either graft removal within the first week after transplantation (n = 2) or primary non-function (n = 9). 74 patients (33%) experienced DGF. Dialysis was initiated prior to Day 3 blood sampling in 89% of patients (n = 65) experiencing DGF. There was no difference between patients with DGF and patients without DGF with respect to donor age, donor’s last P-creatinine, recipient age, gender, baseline P-creatinine, or U-albumin/creatinine-ratio.

**Table 1:** Donor and recipient characteristics.

Donor and recipient characteristics	
Donor age (years) (n = 222)	58 (51–65)
Donor female sex (n = 222)	101 (45%)
Donor’s last P-creatinine (μmol/l) (n = 190)	69 (54–88)
Cold ischemic time (h) (n = 219)	13.5 ±4.4
Recipient age (years) (n = 222)	59 (49–66)
Recipient female sex (n = 222)	88 (40%)
Recipient, preemptive transplantation (n = 222)	40 (18%)
Baseline P-creatinine (μmol/l) (n = 220)	636 (496–756)
Baseline P-NGAL (μg/l) (n = 218)	635 (453–848)
Baseline P-NGAL, preemptive (μg/l) (n = 40)	389 (332–485)
Baseline P-NGAL, on dialysis prior to TX (μg/l) (n = 178)	707 (512–889)
Baseline U-albumin/creatinine-ratio (mg/g) (n = 125)	688 (295–1905)
Baseline U-NGAL (ng/mg) (n = 122)	1784 (698–3924)
Baseline U-L-FABP (ng/mg) (n = 125)	112 (66–181)
Baseline U-cystatin C (mg/g) (n = 122)	15.4 (6.8–26.7)
Baseline U-YKL-40 (ng/mg) (n = 120)	54 (10–175)
Urine output Day 1^a^ (ml/h) (n = 129)	92 (36–158)
Urine output Day 3^a^ (ml/h) (n = 140)	92 (52–140)
Primary non-function (n = 222)	9 (4%)
mGFR Day 5 (ml/min/1.73m^2^) (n = 91)	33 (7–99)
tCr50 (days) (n = 211)	5.8 (1.8–10.9)
DGF^b^ (n = 222)	74 (33%)
mGFR three months (ml/min/1.73m^2^) (n = 148)	43 (34–55)
mGFR twelve months (ml/min/1.73m^2^) (n = 141)	47 (35–60)

Values are mean ±SD, n (%) or median (interquartile range).

^a^Only patients transplanted in Aarhus and Gothenburg.

^b^Excluding patients undergoing graftectomy within the first week after transplantation.

### Effect of remote ischemic conditioning

The intervention (remote ischemic conditioning vs. sham) had no effect on any of the biomarkers at any time point (S2 Fig). Consequently, data was pooled independently from the intervention.

### Effect of variations in the time between reperfusion time and blood and urine sampling

Since blood and urine samples on Day 1 were always collected during daytime working hours on the day after surgery, the time between reperfusion and sampling on Day 1 varied. In order to avoid any potential confounding as a result of this we used information from a subset of Aarhus patients (n = 113 (plasma) and n = 89 (urine)) to analyse biomarker levels in blood and urine depending on the time between reperfusion and sampling on Day 1. No correlation was observed between the elapsed time to Day 1 blood sampling and P-NGAL neither in patients with DGF or in patients without DGF suggesting that biomarker levels were not significantly depending on differences in the time to first day sampling (S3 Fig). Similarly, no significant correlations were observed between the elapsed time to Day 1 sampling and other biomarkers levels (U-NGAL: p = 0.30, p = 0.13; U-cystatin C: p = 0.97, p = 0.23; U-L-FABP: p = 38, p = 0.71; U-YKL-40: p = 0.60, p = 0.24. P-values are for patients with and without DGF respectively).

### P-NGAL, P-creatinine and timed urine output predict DGF

The baseline P-NGAL was higher in patients experiencing DGF and remained elevated on Day 1 and 3 while it decreased in patients that did not require dialysis (S4 Fig). Baseline P-NGAL was approx. 1.8 times higher in patients on dialysis prior to transplantation when compared to patients transplanted preemptive (p<0.001, Table 1). We also found that 40% of the patients on dialysis prior to transplantation (n = 180) experienced DGF, whereas it was only 5% of the preemptive patients (n = 40).

**Fig 2:**
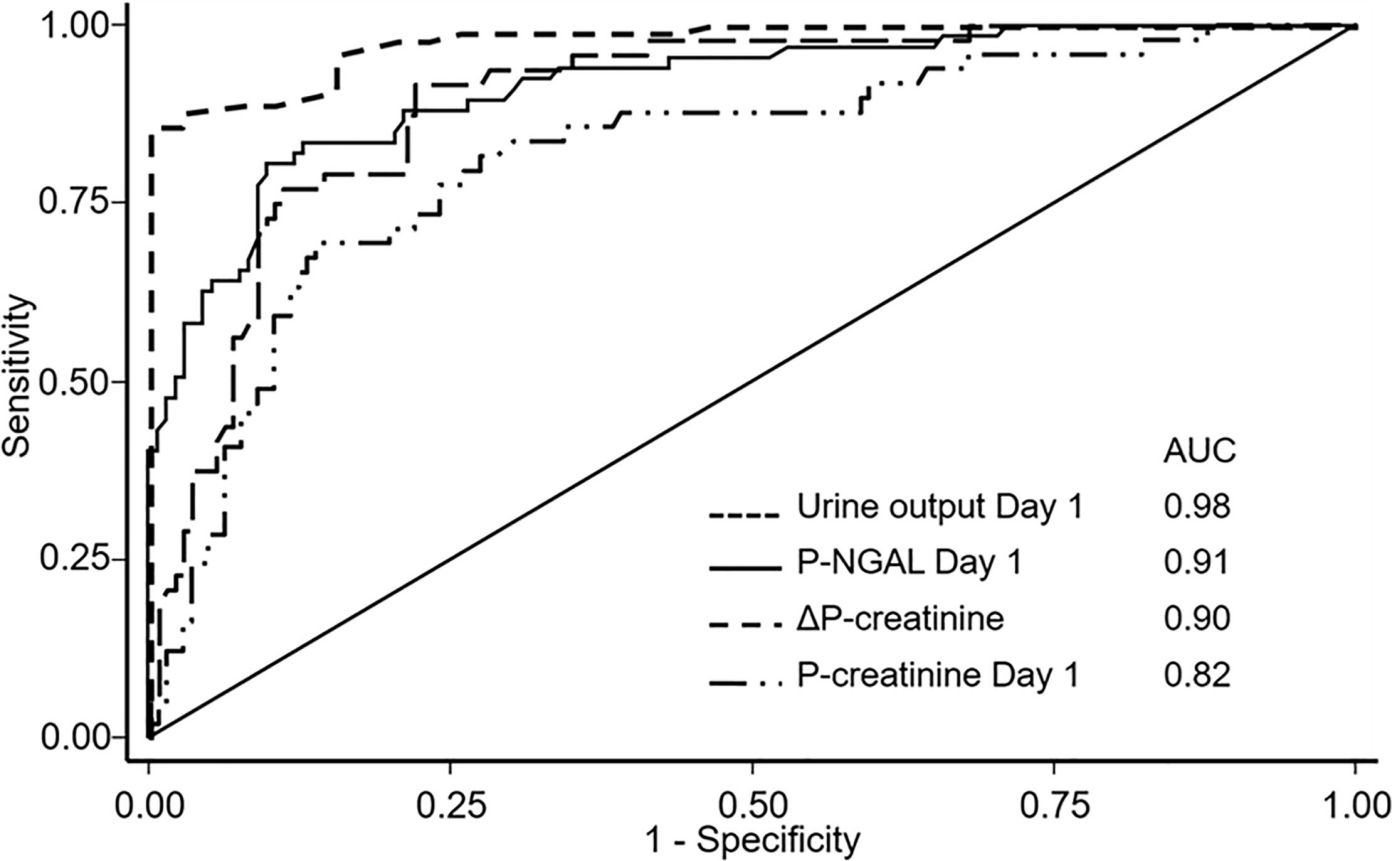
Prediction of DGF. ROC-analyses (AUC) showing the ability to predict DGF for the timed urine output until Day 1 (n = 138), P-NGAL level on Day 1 (n = 199), the change in P-creatinine levels from baseline to Day 1 (n = 194), and P-creatinine level on Day 1 (n = 195).

P-NGAL at Day 1 predicted DGF with a sensitivity of 84% and specificity of 87% (Table 2, Fig 2) and an area under the ROC curve (AUC) of 0.91, and was superior to P-creatinine on Day 1 (p = 0.02) and to the change in P-NGAL from baseline to Day 1 (p<0.001)(Table 2, Fig 2). Patients receiving dialysis prior to P-creatinine sampling on Day 1 were excluded from the latter analysis.

**Table 2:** The ability of biomarkers to predict DGF. AUC = area under the ROC curve. Sens = sensitivity. Spec = specificity. ^a^Change from baseline to Day 1. ^b^Patients receiving dialysis after transplantation but before sampling Day 1 were excluded.^c^Only patients transplanted in Aarhus and Gothenburg.

	Time of sampling after reperfusion	n	AUC ±SE	Optimal cut-off		
				Cut-off	Sens	Spec
ΔP-NGAL^a^	-	176	0.76 ±0.04	-132	0.69	0.74
P-NGAL	90 minutes	208	0.69 ±0.04	614	0.67	0.71
P-NGAL	Day 1	199	0.91 ±0.02	480	0.84	0.87
ΔP-creatinine^a,b^	-	194	0.89 ±0.02	29	0.92	0.78
P-creatinine^b^	Day 1	195	0.82 ±0.04	647	0.69	0.86
Urine output^c^	Day 1	116	0.98 ±0.01	47	0.87	1.00
U-NGAL	90 minutes	136	0.67 ±0.06	1116	0.86	0.48
U-NGAL	Day 1	171	0.82 ±0.04	829	0.79	0.76
U-L-FABP	90 minutes	135	0.52 ±0.07	559	0.33	0.80
U-L-FABP	Day 1	173	0.76 ±0.05	156	0.64	0.87
U-Cystatin C U-Cystatin C	90 minutes	135	0.63 ±0.06	13	0.70	0.49
	Day 1	173	0.73 ±0.05	9	0.65	0.78
U-YKL-40 U-YKL-40	90 minutes	137	0.60 ±0.06	58	0.66	0.50
	Day 1	173	0.78 ±0.04	46	0.85	0.61
U-albumin/creatinine U-albumin/creatinine	90 minutes	137	0.62 ±0.06	2464	0.76	0.48
	Day 1	174	0.84 ±0.04	1365	0.73	0.86

In patients transplanted preemptive P-NGAL at Day 1 predicted DGF with AUC = 0.97 (n = 40) and in patients on dialysis prior to transplantation AUC = 0.90 (n = 162).

A timed urine sample was collected at Day 1 in 58% (n = 129) of the patients enrolled in Aarhus and Gothenburg while nine patients were recorded as being anuric (urine output = 0), allowing 138 patients (62%) for this analysis. In these patients, the urine output sampled at Day 1 was superior to P-creatinine on Day 1 in prediction of DGF (AUC = 0.98 vs 0.80, n = 138), but not to P-NGAL (AUC = 0.94, n = 122; p = 0.07). In 84 (38%) patients no information on urine output was recorded on Day 1.

All urinary biomarkers measured on Day 1 were higher in patients with DGF compared to those with primary function (S4 Fig). U-NGAL and U-albumin/creatinine ratios measured on Day 1 predicted DGF (AUC’s of 0.82 and 0.84, Table 2). However, the biomarkers were inferior in predicting DGF when compared to P-creatinine, P-NGAL or the timed urine output in patients where these were available (S5 Fig). In 13 (6%) patients urinary biomarkers could not be measured due to anuria on Day 1.

### Biomarker levels correlate with early graft function

**Fig 3:**
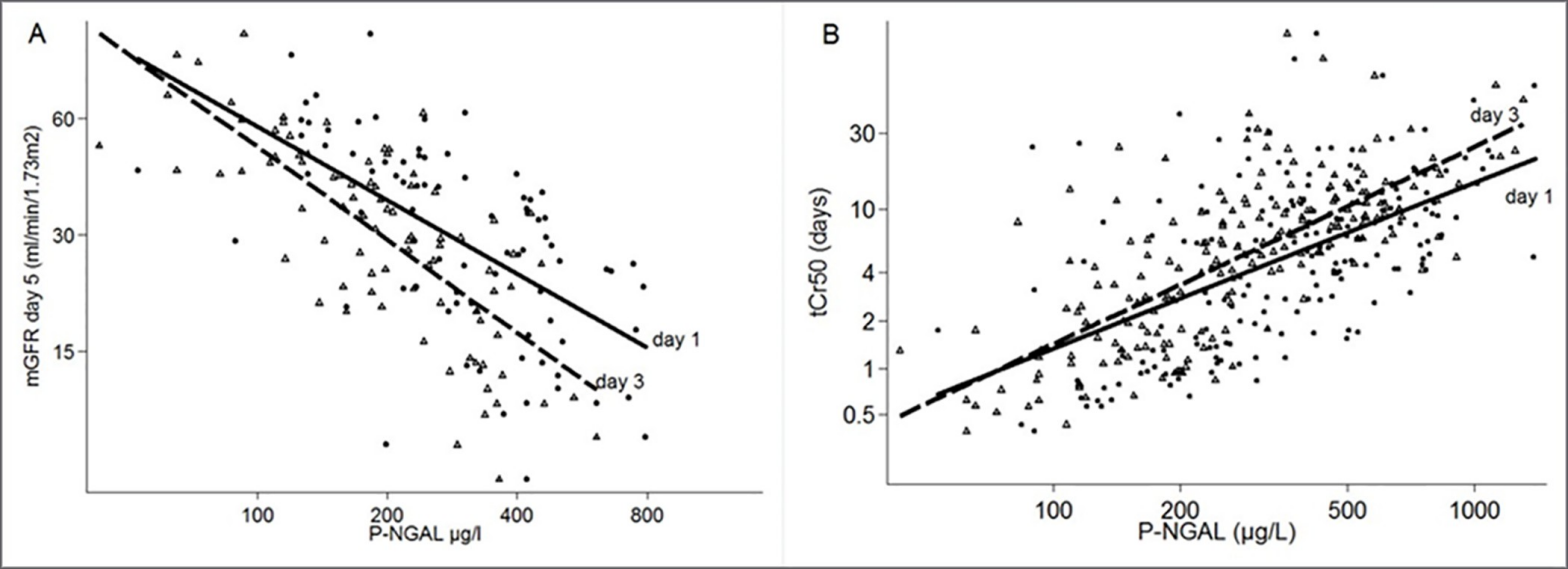
The correlation between P-NGAL levels on Day 1 and 3 and early kidney graft function. A: Correlation between P-NGAL measured on Day 1 (dots) or 3 (triangles) and mGFR at Day 5. B: Correlation between P-NGAL measured on Day 1 (dots) or 3 (triangles) and tCr50.

P-NGAL on both Day 1 and Day 3 correlated with mGFR at Day 5 (r^2^_adj._ = 0.35, p<0.001 and r^2^_adj._ = 0.56, p<0.001) and t50Cr (r^2^_adj._ = 0.31, p<0.001 and r^2^_adj._ = 0.52, p<0.001) (Table 3, Fig 3). mGFR Day 5 was only measured in Aarhus and Gothenburg (n = 91). After adjustment for age, sex, cold ischemic time, intervention, and urine output these correlations remained significant. However, when further adjusted for the change in P-creatinine from baseline to the time of sampling (Day 1 or 3) the correlation was only significant on Day 3 (Table 3). The correlation coefficients for P-NGAL and P-creatinine with respect to mGFR Day 5 or tCr50 were similar for on both Day 1 and 3. In the subset of patients with a recorded urine output on Day 1 this correlated moderately with mGFR Day 5 and tCr50 (Table 3).

**Table 3:** Correlations between P-NGAL, P-creatinine, or urine output and mGFR on Day 5 or tCr50.

	Time of sampling	mGFR Day 5									tCr50		
		Crude			Adjusted^a^			Adjusted^b^			n	p	r^2^_adj._
		n	p	r^2^_adj._	n	p	r^2^_adj._	n	p	r^2^_adj._			
P-NGAL	90 minutes	89	0.45	0.00	60	0.99	0.26	-	-	-			
P-NGAL	Day 1	81	<0.001	0.35	53	0.01	0.35	53	0.55	0.41	192	<0.001	0.31
P-NGAL	Day 3	86	<0.001	0.56	64	<0.001	0.61	64	<0.001	0.63	195	<0.001	0.52
P-creatinine^c^	Day 1	89	<0.001	0.30	60	0.001	0.39	60	0.12	0.45	189	<0.001	0.25
P-creatinine^c^	Day 3	84	<0.001	0.64	67	<0.001	0.66	67	<0.001	0.66	151	<0.001	0.52
Urine output^d^	Day 1	62	<0.001	0.24	60	<0.001	0.30	60	0.25	0.43	104	<0.001	0.24
Urine output^d^	Day 3	69	0.002	0.12	67	0.005	0.16	67	0.09	0.53	117	<0.001	0.13

^a^adjusted for recipient age, recipient sex, cold ischemic time, treatment, and urine output.

^b^adjusted for ^a^ + change in P-creatinine from baseline to time of sampling (Day 1 or 3).

^c^Excluding patients receiving post-transplant dialysis.

^d^Not adjusted for urine output.

U-NGAL, U-cystatin C, U-L-FABP and U-YKL-40 correlated to mGFR on Day 5 and tCr50; however, all correlations were inferior to both P-NGAL and P-creatinine (S1 Table). Similar calculations based on urinary biomarker concentration, without normalization to U-creatinine, did not change the conclusions (S2 Table).

Combining the predictive information from each of the individual, urinary biomarkers using multiple linear regression did not improve the correlations with mGFR on Day 5 or tCr50 (S1 Table). The correlations between U-NGAL alone and mGFR on Day 5 or tCr50 were stronger than any combination of urinary biomarkers.

### Cold ischemic time did not correlate with U-L-FABP

A prolonged cold ischemic time was associated with a lower mGFR on Day 5 and prolonged tCr50 (p = 0.04 and p = 0.01, respectively). U-L-FABP correlated only weakly with cold ischemic time (S6 Fig).

### Early biomarkers do not predict one-year graft function

Only very weak correlations were observed between the biomarkers on Day 1 and graft function at three or twelve months (Table 4). Early urine output did not correlate to any of the one-year graft function parameters.

**Table 4:** Correlations with graft function at three and twelve months. Simple linear regression showing the correlation between biomarkers or urine output measured on Day 1 and kidney graft function (mGFR or eGFR) at three and twelve months.

	Three months						Twelve months					
	mGFR			eGFR			mGFR			eGFR		
	n	p	r^2^_adj._	n	p	r^2^_adj._	n	p	r^2^_adj._	n	p	r^2^_adj._
P-NGAL	135	0.05	0.02	188	0.07	0.01	128	0.02	0.04	180	0.07	0.01
U-NGAL	117	0.99	-0.01	163	0.62	0.00	114	0.22	0.00	156	0.13	0.01
U-L-FABP	119	0.81	-0.01	165	0.30	0.00	115	0.97	-0.01	158	0.46	0.00
U-cystatin C	119	0.27	0.00	165	0.24	0.00	116	0.04	0.03	158	0.06	0.02
U-YKL-40	119	0.22	0.00	165	0.02	0.03	115	0.02	0.04	158	0.03	0.02
Urine output	86	0.05	0.04	121	0.49	0.00	79	0.15	0.01	116	0.81	-0.01

## Discussion

This study has identified a strong correlation between P-NGAL measured on Day 1 and the early kidney graft function. Furthermore, P-NGAL predicted DGF with acceptable sensitivity and specificity. Urinary biomarkers, either individually or combined were only weakly correlated to the initial graft function and DGF. In 62% of the patients, a 24hr urine output was recorded on Day 1. In these patients, P-NGAL was not superior to the urine output in predicting DGF, but the high number of missing samples limits the interpretation of this.

The finding that P-NGAL on Day 1 performed better when predicting DGF than P-creatinine on Day 1 is consistent with previous studies[3,20,32]. The AUC of ΔP-crea was similar to the AUC of P-NGAL suggesting that the change in P-creatinine within the first day may be as predictive as P-NGAL on Day 1. Interestingly, baseline P-NGAL prior to reperfusion was elevated in patients who experienced DGF. This may indicate that recipient dependent factors may affect both P-NGAL and an increased risk of DGF. Higher baseline levels were observed in patients on dialysis prior to transplantation and these patients had as expected a higher risk of experiencing DGF than patients transplanted preemptive. This may partly depend on the residual function of the kidney. Unfortunately, data on residual function was not available in this cohort. Nevertheless, P-NGAL on Day 1 also predicted DGF in this subgroup.

None of the biomarkers correlated well with graft function at three or twelve months post-transplant. A review identified only one study showing that U-NGAL on Day 4 and Day 7 was associated with serum creatinine twelve months after kidney transplantation whereas the remaining, included studies found no association [22].

All urinary biomarkers, including U-albumin/creatinine ratio, correlated poorly with mGFR on Day 5 and tCr50 when compared to P-NGAL or P-creatinine. Their ability to predict DGF was also poorer than P-NGAL, P-creatinine or urine output. Two previous studies showed that U-NGAL on Day 2 predicted DGF better than P-creatinine, but no better than urine output [24,33]. Both studies were smaller with 40 and 170 transplants patients respectively. In contrast to these studies we measured the biomarkers in spot urine samples normalized to U-creatinine levels[15]. This lead to different results as GFR and thus the urine creatinine excretion rate is not in steady state immediately after kidney transplantation[34]. In addition, the inter-individual variation in muscle mass and possible muscle injury associated with surgery may also affect U-creatinine. Model calculations[34] and a previous study[2] have suggested that normalization to U-creatinine may overestimate the biomarker excretion rate; however, in our study the ability of these biomarkers to predict DGF was not improved when recalculated using urinary biomarker concentration rather than the ratio to U-creatinine. In contrast to previous studies[1,35] the combination of several urinary biomarkers using multiple regression analysis did not improve the correlation of urinary biomarkers with mGFR on Day 5 or tCr50. Our findings thus suggest that even though the urinary biomarkers may be pertinent in ischemia-reperfusion injury, their ability to predict early graft function and DGF is poor in a clinical setting and in part be affected by missing data mainly due to anuria.

The strength of this study is that a large, multicenter study on renal biomarkers in kidney transplantation. Moreover, the patients in the study represent an unselected population of deceased donor kidney transplant recipients. Our findings may be affected by the limitations associated with normalizing urinary biomarkers as mentioned above. Measuring the biomarker excretion rate in a 24hr urine sample may prove more sensitive. However, this would not only delay the measurements, but also be time consuming and possibly impractical in clinical practice. In this study the collection of timed urine samples was in fact only possible in a subset of the patients included in Aarhus and Gothenburg. Due to the clinical practice of routine blood and urine collection during daytime, the time between reperfusion and blood or urine sampling on Day 1 varied. In principle, this may cause additional variation in Day 1 biomarker levels and reduce sensitivity and specificity. However, we did not identify any systematic difference between the time interval and biomarker levels neither in patients with or patients without DGF indicating that this did not significantly affect the results. Furthermore, the sampling procedure reflects the clinical practice in which biomarkers would have to be applied.

In conclusion, P-NGAL measured on Day 1 post-transplant predicts DGF after deceased donor kidney transplantation and correlates with early graft function, while the urinary biomarkers U-NGAL, U-L-FABP, U-cystatin C, and U-YKL-40 correlated poorly and may not be useful for predicting DGF. The urine output on Day 1 was more accurate than P-creatinine and P-NGAL in predicting DGF; however, this is limited by the fact that a timed urine volume was only measured in 62% of the patients. None of the biomarkers measured on Day 1 were useful for predicting graft function at three or twelve months.

## Supporting information

S1 Fig Time of sampling. Timeline showing the time points of mGFR measurements as well as blood and urine sampling.
(PDF)

S2 Fig Urinary biomarkers and intervention. Biomarker levels (A: P-NGAL; B: U-NAGL; C: U-LABP, D: U-Cystatin C, E: U-YKL-40) depending on the intervention (ischemic conditioning (rIC)). No differences are observed in relation to intervention. White boxes = non-rIC. Black boxes = rIC.
(PDF)

S3 Fig P-NGAL and elapsed time. P-NGAL measured on Day 1 as a function of the elapsed time between kidney graft reperfusion and blood sampling. There is no significant correlation between P-NGAL-level and the time elapsed between reperfusion and first sampling for patients without DGF (●) and only a very weak, negative correlation for patients with DGF (x).
(PDF)

S4 Fig Biomarkers and DGF. Time dependent changes of biomarkers levels depending on the presence of DGF or no DGF. A significant difference between P-NGAL, U-NGAL, U-Cystatin C and U-YKL-40, but not U-LABP, was observed at Day 1 and 3 after transplantation. Black boxes: DGF. White boxes: no DGF after transplantation.
(PDF)

S5 Fig Urinary biomarkers and DGF prediction. ROC-analyses showing the ability of the urinary biomarkers on day 1 to predict DGF after transplantation.
(PDF)

S6 Fig Cold ischemia time and U-L-FABP. The correlation between cold ischemia time and U-L-FABP at 90 min and 1 day after reperfusion. Only a very weak correlation was identifed (90 min: r = -0.20; p = 0.02; Day 1: r = 0.22; p = 0.003).
(PDF)

S1 Table Urinary biomarkers and kidney graft function. The correlation between urinary biomarker levels and mGFR at Day 5 or tCr50. Only weak correlations were observed between selected biomarkers at various sampling points and mGFR at Day 5 or tCr50. ^a^U-NGAL, U-L-FABP, U-cystatin C, U-YKL-40 and U-albumin combined using multiple linear regression. All biomarkers were normalized to U-creatinine.
(PDF)

S2 Table The correlations of the urinary biomarkers and mGFR at Day 5 or tCr50 without normalization of the biomarkers to U-creatinine. (PDF)

S1 Document Protocol of the CONTEXT study in Danish. (PDF)

S2 Document Comments to the CONSORT checklist. (DOC)
