# Correction: P-NGAL Day 1 predicts early but not one year graft function following deceased donor kidney transplantation–The CONTEXT study

**DOI:** 10.1371/journal.pone.0304722

**Published:** 2024-05-29

**Authors:** Marie B. Nielsen, Nicoline V. Krogstrup, Gertrude J. Nieuwenhuijs-Moeke, Mihai Oltean, Frank J. M. F. Dor, Bente Jespersen, Henrik Birn

Reference 31 is incorrect. The correct reference is: Levey AS, Coresh J, Greene T, Stevens LA, Zhang Y, Hendriksen S, et al. Using Standardized Serum Creatinine Values in the Modification of Diet in Renal Disease Study Equation for Estimating Glomerular Filtration Rate. 2006.

There is an error in Table 4. The estimated glomerular filtration (eGFR) values were estimated using an incorrect version of the MDRD-formula. Please see the correct Table 4 here.

**Table 4:** Correlations with graft function at three and twelve months. Simple linear regression showing the correlation between biomarkers or urine output measured on Day 1 and kidney graft function (mGFR or eGFR) at three and twelve months.

	Three months						Twelve months					
	mGFR			eGFR			mGFR			eGFR		
	n	p	r^2^_adj._	n	p	r^2^_adj._	n	p	r^2^_adj._	n	p	r^2^_adj._
P-NGAL	135	0.05	0.02	188	0.11	0.01	128	0.02	0.04	180	0.10	0.01
U-NGAL	117	0.99	-0.01	163	0.90	-0.01	114	0.22	0.00	156	0.27	0.01
U-L-FABP	119	0.81	-0.01	165	0.56	0.00	115	0.97	-0.01	158	0.46	-0.01
U-cystatin C	119	0.27	0.00	165	0.43	0.00	116	0.04	0.03	158	0.15	0.01
U-YKL-40	119	0.22	0.00	165	0.03	0.02	115	0.02	0.04	158	0.05	0.02
Urine output	86	0.05	0.04	121	0.50	0.00	79	0.15	0.01	116	0.83	-0.01

## References

[1] Nielsen MB, Krogstrup NV, Nieuwenhuijs-Moeke GJ, Oltean M, Dor FJMF, Jespersen B, et al. (2019) P-NGAL Day 1 predicts early but not one year graft function following deceased donor kidney transplantation–The CONTEXT study. PLoS ONE 14(2): e0212676. 10.1371/journal.pone.0212676 30817778 PMC6394926

