# Supplementary material for: P-NGAL Day 1 predicts early but not one year graft function following deceased donor kidney transplantation – The CONTEXT study
Source: PLoS One. 2019 Feb 28;14(2):e0212676. doi: 10.1371/journal.pone.0212676 (PMC6394926; doi:10.1371/journal.pone.0212676)

Supporting information Figure 1

Timeline showing the time points of mGFR measurements as well as blood and urine sampling.

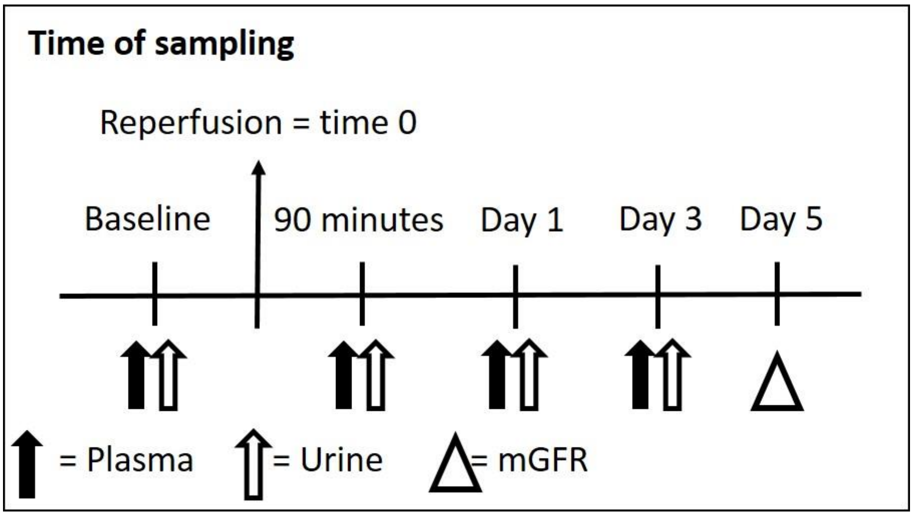

Supplement: S1 Fig — Timeline showing the time points of mGFR measurements as well as blood and urine sampling. (PDF) [file pone.0212676.s001.pdf]
