# Supplementary material for: P-NGAL Day 1 predicts early but not one year graft function following deceased donor kidney transplantation – The CONTEXT study
Source: PLoS One. 2019 Feb 28;14(2):e0212676. doi: 10.1371/journal.pone.0212676 (PMC6394926; doi:10.1371/journal.pone.0212676)

Supporting information Figure 2

Biomarker levels (A: P-NGAL; B: U-NAGL; C: U-LABP, D: U-Cystatin C, E: U-YKL-40) depending on the intervention (ischemic conditioning (rIC)). No differences are observed in relation to intervention. White boxes=non-rIC. Black boxes=rIC.

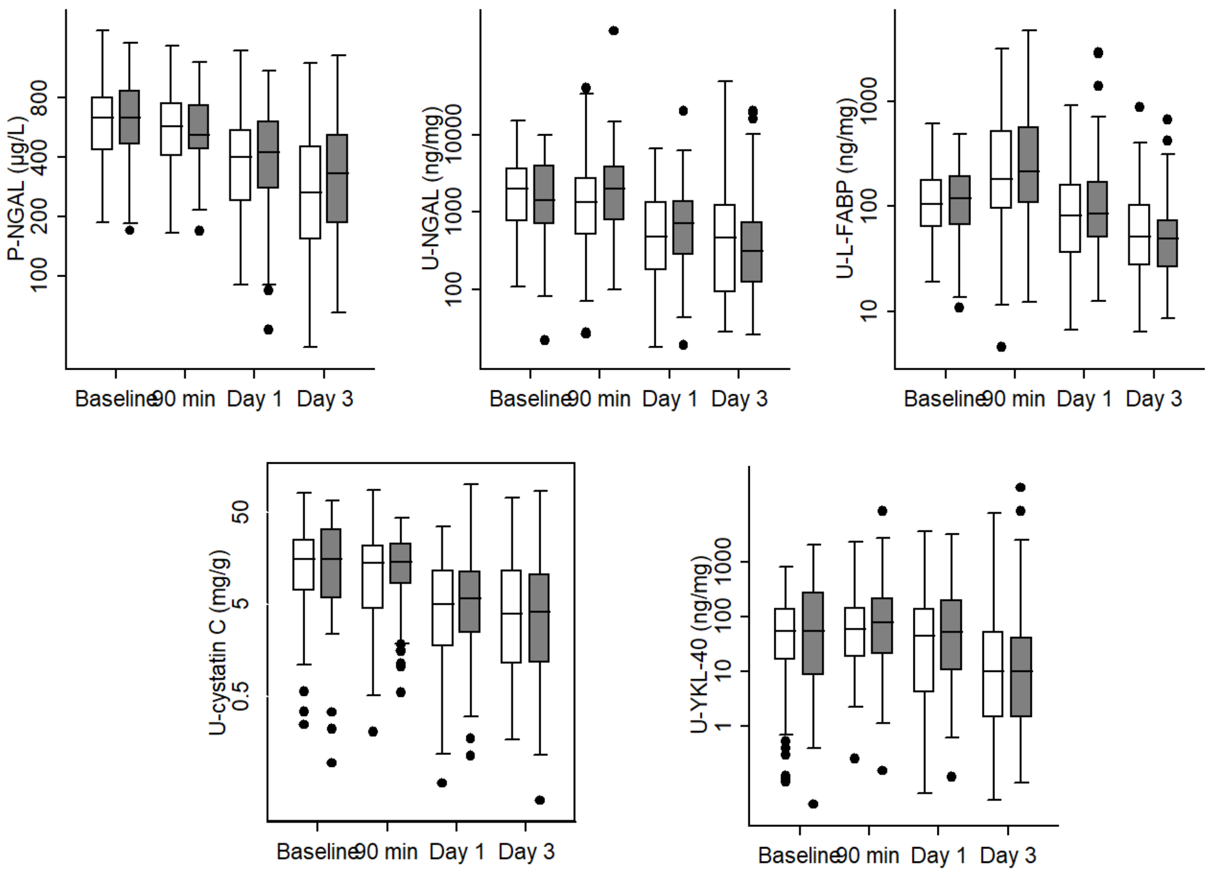

Supplement: S2 Fig — Biomarker levels (A: P-NGAL; B: U-NAGL; C: U-LABP, D: U-Cystatin C, E: U-YKL-40) depending on the intervention (ischemic conditioning (rIC)). No differences are observed in relation to intervention. White boxes = non-rIC. Black boxes = rIC. (PDF) [file pone.0212676.s002.pdf]
