# Supplementary material for: P-NGAL Day 1 predicts early but not one year graft function following deceased donor kidney transplantation – The CONTEXT study
Source: PLoS One. 2019 Feb 28;14(2):e0212676. doi: 10.1371/journal.pone.0212676 (PMC6394926; doi:10.1371/journal.pone.0212676)

Supporting information Figure 3

P-NGAL measured on Day 1 as a function of the elapsed time between kidney graft reperfusion and blood sampling. There is no significant correlation between P-NGAL-level and the time elapsed between reperfusion and first sampling for patients without DGF (●) and only a very weak, negative correlation for patients with DGF (x).

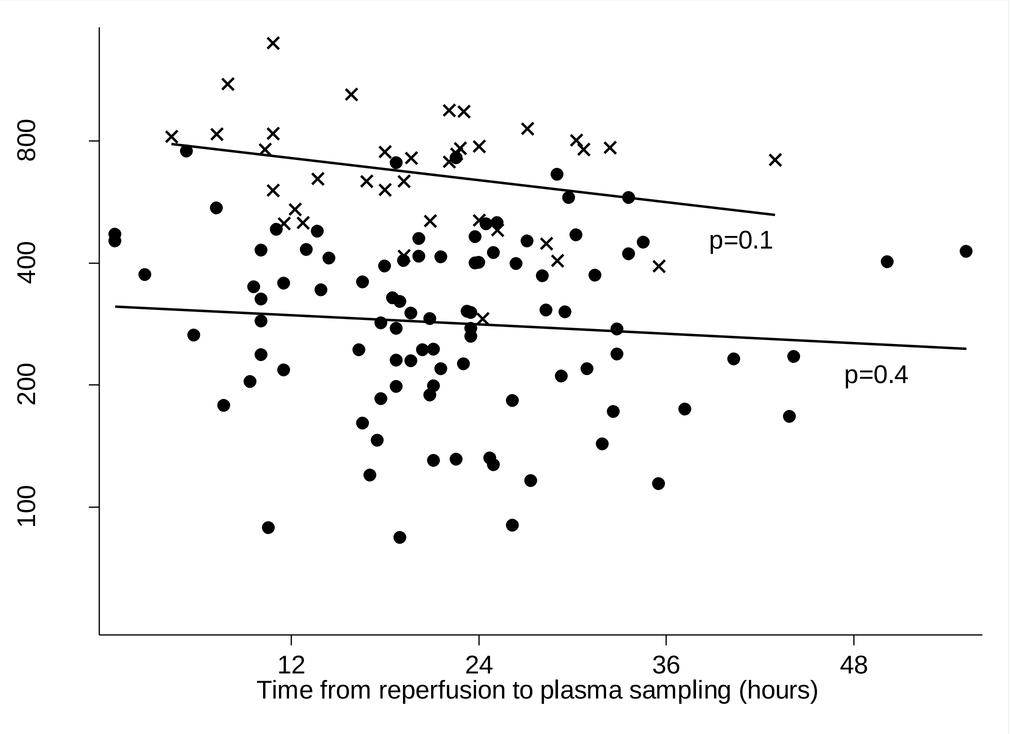

Supplement: S3 Fig — P-NGAL measured on Day 1 as a function of the elapsed time between kidney graft reperfusion and blood sampling. There is no significant correlation between P-NGAL-level and the time elapsed between reperfusion and first sampling for patients without DGF (●) and only a very weak, negative correlation for patients with DGF (x). (PDF) [file pone.0212676.s003.pdf]
