# Supplementary material for: P-NGAL Day 1 predicts early but not one year graft function following deceased donor kidney transplantation – The CONTEXT study
Source: PLoS One. 2019 Feb 28;14(2):e0212676. doi: 10.1371/journal.pone.0212676 (PMC6394926; doi:10.1371/journal.pone.0212676)

Supporting information Figure 4

Time dependent changes of biomarkers levels depending on the presence of DGF or no DGF. A significant difference between P-NGAL, U-NGAL, U-Cystatin C and U-YKL-40, but not U-LABP, was observed at Day 1 and 3 after transplantation. Black boxes: DGF. White boxes: no DGF after transplantation.

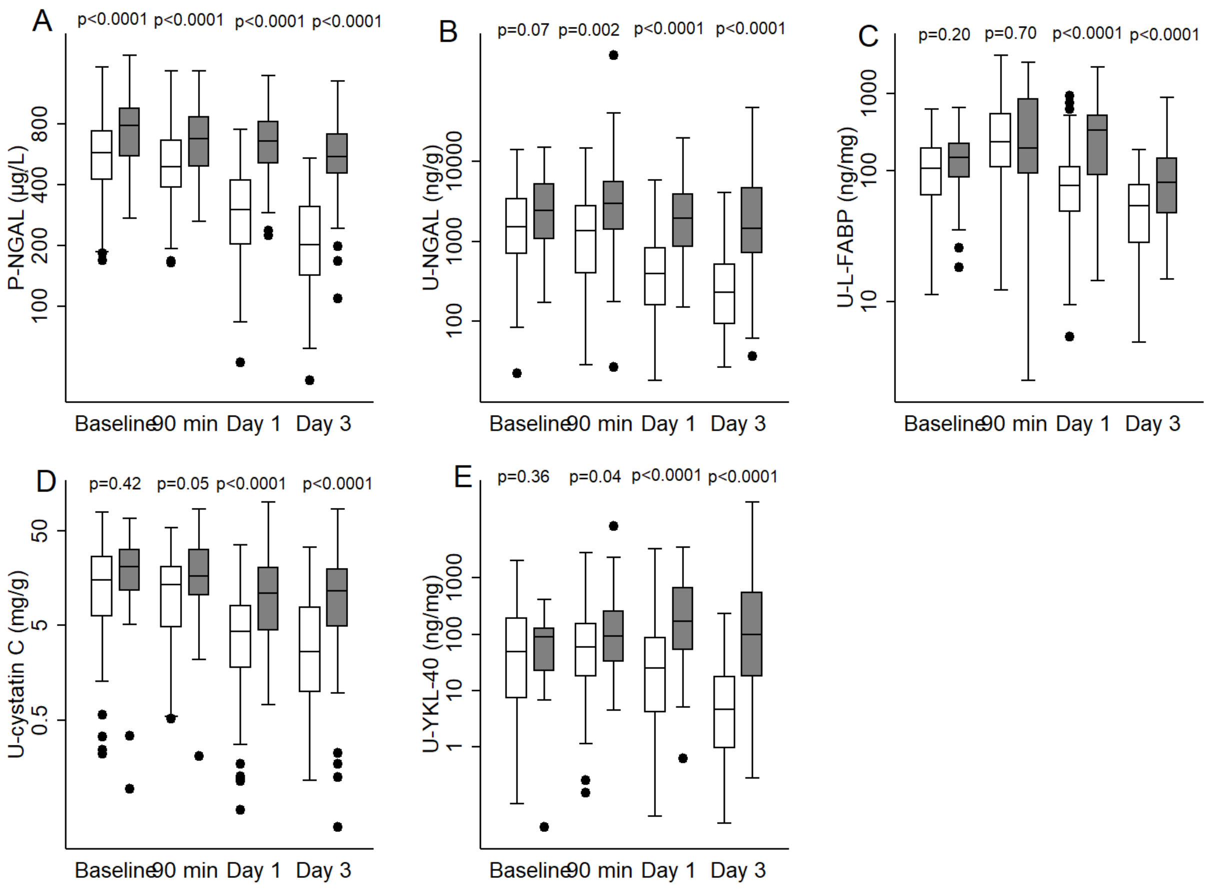

Supplement: S4 Fig — Time dependent changes of biomarkers levels depending on the presence of DGF or no DGF. A significant difference between P-NGAL, U-NGAL, U-Cystatin C and U-YKL-40, but not U-LABP, was observed at Day 1 and 3 after transplantation. Black boxes: DGF. White boxes: no DGF after transplantation. (PDF) [file pone.0212676.s004.pdf]
