# Supplementary material for: P-NGAL Day 1 predicts early but not one year graft function following deceased donor kidney transplantation – The CONTEXT study
Source: PLoS One. 2019 Feb 28;14(2):e0212676. doi: 10.1371/journal.pone.0212676 (PMC6394926; doi:10.1371/journal.pone.0212676)

## Supporting information Figure 5

ROC-analyses showing the ability of the urinary biomarkers on day 1 to predict DGF after transplantation.

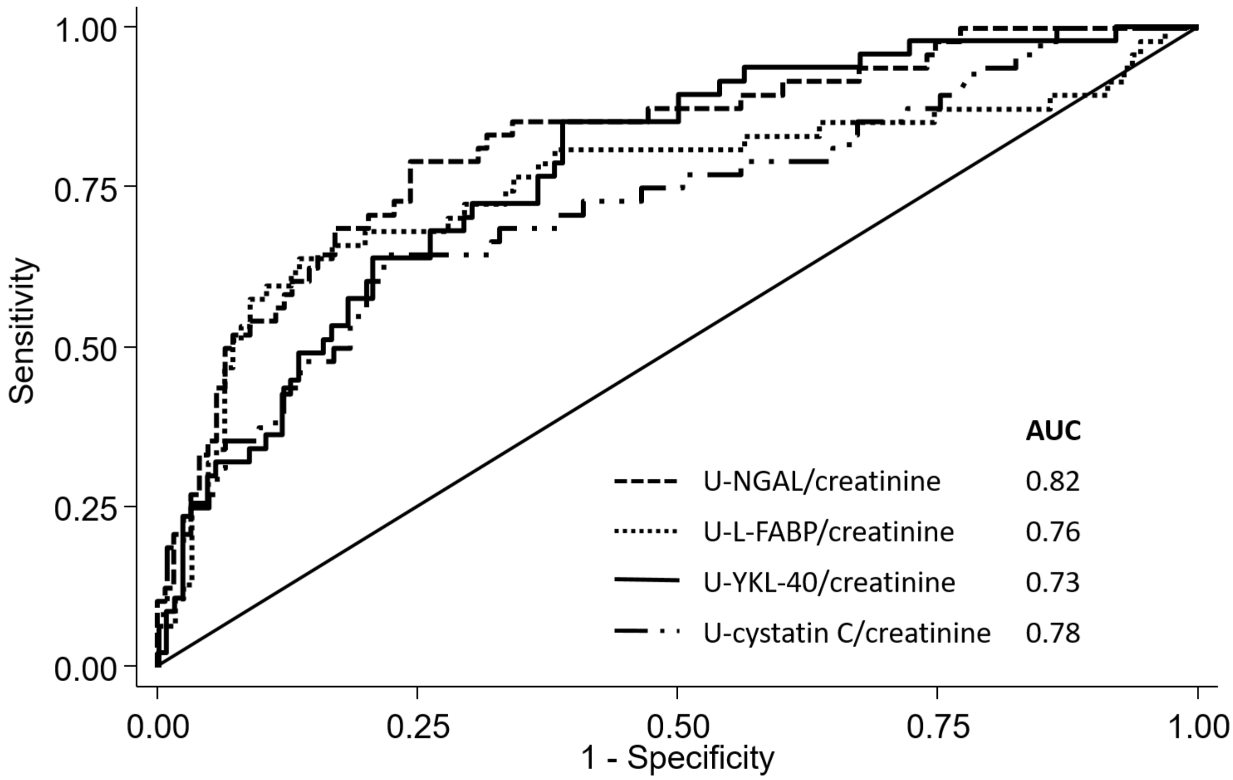

Supplement: S5 Fig — ROC-analyses showing the ability of the urinary biomarkers on day 1 to predict DGF after transplantation. (PDF) [file pone.0212676.s005.pdf]
