# Supplementary material for: P-NGAL Day 1 predicts early but not one year graft function following deceased donor kidney transplantation – The CONTEXT study
Source: PLoS One. 2019 Feb 28;14(2):e0212676. doi: 10.1371/journal.pone.0212676 (PMC6394926; doi:10.1371/journal.pone.0212676)

Supporting information Figure 6

The correlation between cold ischemia time and U-L-FABP at 90 min and 1 day after reperfusion. Only a very weak correlation was identifed (90 min: r=-0.20; p=0.02; Day 1: r=0.22; p=0.003).

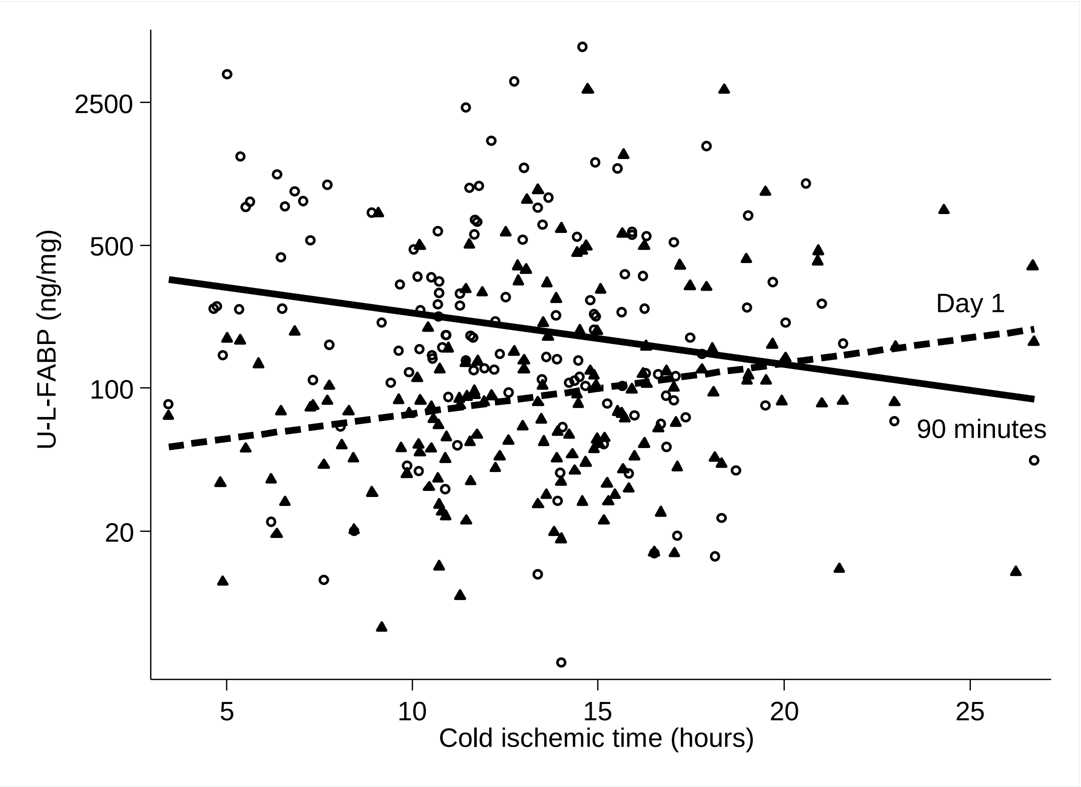

Supplement: S6 Fig — The correlation between cold ischemia time and U-L-FABP at 90 min and 1 day after reperfusion. Only a very weak correlation was identifed (90 min: r = -0.20; p = 0.02; Day 1: r = 0.22; p = 0.003). (PDF) [file pone.0212676.s006.pdf]
