# Supplementary material for: P-NGAL Day 1 predicts early but not one year graft function following deceased donor kidney transplantation – The CONTEXT study
Source: PLoS One. 2019 Feb 28;14(2):e0212676. doi: 10.1371/journal.pone.0212676 (PMC6394926; doi:10.1371/journal.pone.0212676)
Supplement: S1 Table — The correlation between urinary biomarker levels and mGFR at Day 5 or tCr50. Only weak correlations were observed between selected biomarkers at various sampling points and mGFR at Day 5 or tCr50. aU-NGAL, U-L-FABP, U-cystatin C, U-YKL-40 and U-albumin combined using multiple linear regression. All biomarkers were normalized to U-creatinine. (PDF) [file pone.0212676.s007.pdf]

## Supporting information Table 1

The correlation between urinary biomarker levels and mGFR at Day 5 or tCr50. Only weak correlations were observed between selected biomarkers at various sampling points and mGFR at Day 5 or tCr50. [a]U-NGAL, U-L-FABP, U-cystatin C, U-YKL-40 and U-albumin combined using multiple linear regression. All biomarkers were normalized to U-creatinine.

| | Time of sampling | mGFR day 5 | | | tCr50 | | |
|---|---|---|---|---|---|---|---|
| | | n | p | $r^2_{adj.}$ | n | p | $r^2_{adj.}$ |
| U-NGAL (ng/mg) | 90 minutes | 73 | 0.02 | 0.06 | 132 | 0.23 | 0.00 |
| | Day 1 | 72 | <0.001 | 0.19 | 166 | <0.001 | 0.18 |
| | Day 3 | 73 | <0.001 | 0.15 | 161 | <0.001 | 0.20 |
| U-L-FABP (ng/mg) | 90 minutes | 73 | 0.13 | 0.02 | 132 | 0.51 | 0.00 |
| | Day 1 | 74 | 0.40 | 0.00 | 168 | <0.001 | 0.07 |
| | Day 3 | 75 | 0.97 | -0.01 | 161 | 0.005 | 0.04 |
| U-cystatin C (mg/g) | 90 minutes | 73 | 0.10 | 0.02 | 132 | 1.00 | 0.00 |
| | Day 1 | 73 | 0.01 | 0.09 | 168 | <0.001 | 0.08 |
| | Day 3 | 76 | <0.001 | 0.17 | 162 | <0.001 | 0.15 |
| U-YKL-40 (ng/mg) | 90 minutes | 73 | 0.16 | 0.01 | 133 | 0.54 | 0.00 |
| | Day 1 | 74 | 0.01 | 0.07 | 168 | <0.001 | 0.15 |
| | Day 3 | 74 | 0.13 | 0.02 | 160 | <0.001 | 0.16 |
| U-albumin/creatinine (mg/g) | 90 minutes | 73 | 0.94 | -0.01 | 133 | 0.84 | 0.00 |
| | Day 1 | 74 | 0.01 | 0.07 | 169 | <0.001 | 0.17 |
| | Day 3 | 76 | 0.42 | 0.00 | 164 | <0.001 | 0.10 |
| U-biomarkers combined[a] | Day 1 | 72 | 0.003 | 0.19 | 165 | 0.001 | 0.25 |
| | Day 3 | 71 | 0.57 | 0.26 | 155 | 0.24 | 0.19 |
