# Supplementary material for: P-NGAL Day 1 predicts early but not one year graft function following deceased donor kidney transplantation – The CONTEXT study
Source: PLoS One. 2019 Feb 28;14(2):e0212676. doi: 10.1371/journal.pone.0212676 (PMC6394926; doi:10.1371/journal.pone.0212676)
Supplement: S2 Table — (PDF) [file pone.0212676.s008.pdf]

Supporting information Table 2

The correlations of the urinary biomarkers and mGFR at Day 5 or tCr50 without normalization of the biomarkers to U-creatinine.

	Time of sampling	mGFR Day 5			tCr50		
		n	p	$r^2_{adj.}$	n	p	$r^2_{adj.}$
U-NGAL (µg/L)	90 minutes	75	0.14	0.01	137	0.57	-0.01
	Day 1	72	<0.001	0.17	166	<0.00.1	0.13
	Day 3	73	<0.001	0.16	161	<0.001	0.21
U-L-FABP (ng/ml)	90 minutes	74	0.73	-0.01	136	0.12	0.01
	Day 1	74	0.46	-0.01	168	0.05	0.02
	Day 3	76	0.93	-0.01	162	0.01	0.04
U-Cystatin C (mg/L)	90 minutes	74	0.70	-0.01	136	0.23	0.00
	Day 1	73	0.01	0.09	168	0.01	0.04
	Day 3	76	<0.001	0.20	162	<0.001	0.18
U-YKL-40 (pg/ml)	90 minutes	74	0.50	-0.01	137	0.83	-0.01
	Day 1	74	0.01	0.07	168	<0.001	0.12
	Day 3	75	0.11	0.02	161	<0.001	0.17
