# Supplementary material for: P-NGAL Day 1 predicts early but not one year graft function following deceased donor kidney transplantation – The CONTEXT study
Source: PLoS One. 2019 Feb 28;14(2):e0212676. doi: 10.1371/journal.pone.0212676 (PMC6394926; doi:10.1371/journal.pone.0212676)
Supplement: S1 Document — (PDF) [file pone.0212676.s009.pdf]

Projektbeskrivelse til klinisk forsøg

# CONTEXT
## Remote Ischemic Conditioning in Renal Transplantation – Effect on Immediate and Extended Kidney Graft Function.

-udnyttelse af kroppens naturlige værn mod iltmangel til forebyggelse af akut nyresvigt ved nyretransplantation

**Projektgruppe**
Nicoline V. Krogstrup, læge[1]
Henrik Birn, professor, dr. med.[2]
Rikke Nørregaard, cand. scient., ph.d. [1]
Ulla Møldrup, overlæge[3]
Øyvind Østraat, overlæge dr. med.[3]
Bente Jespersen, professor, overlæge dr. med. [2]

1.: Klinisk Institut, Aarhus Universitetshospital, Skejby, 2.: Nyremedicinsk afd. C, Aarhus Universitetshospital, Skejby, 3.: Urologisk afd. K, Aarhus Universitetshospital, Skejby

**Forsøgsansvarlig:**
Nicoline V. Krogstrup, læge, Klinisk Institut, Aarhus Universitetshospital, Skejby, Brendstrupgårdsvej 100, 8200 Århus N, e-mail: nicoline.v.krogstrup@ki.au.dk, Tlf: 78 45 24 17 / 61 26 81 23
Sundhedsstyrrelsens autorisations ID: 06RVV

## BAGGRUND

Shocknyre er en hyppig komplikation til nyretransplantation væsentligst betinget af iskæmi (iltmangel i væv) og reperfusionsskader i forbindelse med udtagning og indsættelse af nyren. Shocknyre ses hos 25% af organmodtagere (recipienter) ved nyretransplantation fra en hjernedød donor og viser sig oftest ved manglende funktion af graften (den transplanterede nyre) straks efter transplantationen, også kaldet delayed graft function (DGF). Dette kan føre til forlænget behov for dialyse, som er belastende for patienten og forbundet med store økonomiske omkostninger. Samtidig øges risikoen for afstødning (rejektion) (1), medførende risiko for vedvarende forringet funktion og nedsat levetid af nyren.
Endotelcellerne, der beklæder blodkar, er det umiddelbare mål for den inflammatoriske reaktion som initieres efter iskæmi/reperfusion, og beskadigelse af disse celler resulterer i forsnævret karlumen og reduceret blodflow. Samtidig aktiveres endotelcellerne, så dendritiske celler (DC) forankres og vandrer over karvæggen. DC initierer det immunologiske respons, idet de optager alloantigener fra graften, migrerer videre til det lymfoide væv og iværksætter et T-celle-medieret immunorespons (2) rettet mod den nye nyre, hvilket nødvendiggør intensiveret immundæmpende behandling, fremskynder nedbrydning/fibrosedannelse i graften og i de alvorligste tilfælde kan føre til akut tab af nyren.
Nyere undersøgelser gennemført af vores samarbejdspartnere på Århus Universitetshospital, Skejby, har netop vist, at gentagen, kortvarig iskæmi i overarmen (rIC) induceret ved opblæsning af en blodtryksmanchet under transport til hospitalet kan aktivere selvbeskyttelse mod iskæmi i hjertet, så en mindre del af hjertemusklen tager varig skade ved blodprop i hjertet (3,4). Fremgangsmåden har også vist sig at kunne forebygge iskæmi i hjertet blandt børn opereret for medfødte hjertemisdannelser (5). I grisemodeller øges flow i koronarkarrene (6), og ved hjertetransplantation fra hjernedøde grisedonorer reducerer rIC på recipienten iskæmi og reperfusionsskader i det transplanterede hjerte (7). Humane pilotstudier har vist, at rIC kan beskytte mod shocknyre efter elektiv operation for aortaaneurisme (8), og vi undersøger i øjeblikket effekten af rIC mod nyrefunktionspåvirkning efter hjerteoperation blandt børn.
Der er forventninger om, at rIC kan beskytte mod shocknyre efter nyretransplantation (9,10), og vores resultater fra en grisemodel med transplantation fra hjernedød donor viser at rIC udført på recipienten gav højere tidlig GFR (11). Samtidig sås en bedre blodgennemstrømning af nyrerne bedømt ved MR scanning. Der er endnu ikke gennemført humane undersøgelser.
Mekanismen bag vævsbeskyttende effekt af rIC er ikke fuldstændigt forstået, men det formodes, at mediatorer fra det prækonditionerede område forbigående inducerer syntesen af hypoxia inducible factor i celler (12-14) hvorved vævet, herunder endotelet i graften, beskyttes mod skader og adhæsion af immunkompetente celler – primært DC (15,16). Det er planen at identificere og kvantificere DC i blod ved flowcytometri, idet forekomst af DC i blodet menes at afspejle DC aktivering af betydning for udvikling af rejektion (17). Flere studier har identificeret ændringer i urinudskillelse af shocknyremarkører ved akut, iskæmisk nyreskade, og vi agter derfor at måle urinudskillelsen af en række af disse, bl.a. neutrophil gelatinase-associated lipocalin (NGAL) (18) og 8-isoprostane (19), de første døgn efter transplantation og vurdere deres evne til at forudsige senere funktion af graftnyren og effekt af rIC, idet vi kan måle NGAL i eget laboratorium og i grisemodellen kunne vise, at rIC førte til lavere NGAL udskillelse i urinen samtidig med højere glomerulær filtrationshastighed (GFR).

## FORMÅL

Det planlagte studie skal vise, om rIC af recipienten af en transplanteret nyre kan forbedre den tidlige nyregraftfunktion og mindske risikoen for shocknyre, modulere det tidlige immunologiske

respons og modvirke tendensen til rejektion af nyregraften. Ligeledes undersøges om rIC øger nyrefunktionen på længere sigt.
Viser det sig at rIC giver en nyrebeskyttende effekt vil det have stor betydning for kommende patienter samt give store økonomiske besparelser.

## HYPOTESER

1) Remote ischemic conditioning (rIC) udført på recipienten af en nyre fra afdød donor kan bedre tidlig nyrefunktion vurderet ved ændringer i plasma kreatinin, behovet for dialyse den første uge, samt GFR og renografi (vurdering af blodgennemstrømning og tubulusfunktion) målt med isotopteknik 5 dage efter transplantationen.
2) rIC kan forhindre iskæmiske skader i en transplanteret nyre hvilket afspejles i a) færre strukturelle ændringer i nyrevæv udtaget ved biopsi 6 dage efter transplantationen, b) mindre stigning i biomarkører målt i blod og urin og c) modulation af det tidligere immunologiske respons vurderet ved ændringer i relevante markører, herunder DC 6 dage efter transplantationen.
3) nyrefunktionen bedømt ved GFR er bedre et år efter transplantationen, når recipienten af en nyre fra afdød donor har været forbehandlet med rIC

## TIDSPERIODE

Inklusion forventes påbegyndt 01.04.2011 og afsluttet 31.03.2013. Observationsperioden forventes afsluttet 31.03.2014.

## PROTOKOL

**Design**. Investigatorinitieret, randomiseret, kontrolleret, blindet studie.
**Statistik.** Styrkeberegninger udført i samråd med statistiker og baseret på effekten af rIC på den umiddelbare graftfunktion vurderet ved ændringshastigheden i p-creatinin viser, at det vil være nødvendigt at inkludere 200 patienter, der modtager nyre fra en afdød.
**Patienter.** Myndige patienter, der gennemgår transplantation med nyre fra hjernedød donor ved nyremedicinsk afdeling C, Århus Universitetshospital, Skejby, eller ved Transplantationsenheden Sahlgrenska i Göteborg, Sverige. Der transplanteres i alt cirka 150 patienter årligt med nyre fra hjernedød donor på de to centre, og det forventes at inkludere 200 patienter i forsøget.

*Inklusionskriterier:*

1) >18 år og myndig
2) Informeret mundtligt og skriftlig samtykke
3) Kandidat til nekronyretransplantation

*Ekslusionskriterier:*

1) Ude af stand til at afgive informeret samtykke
2) AV-fistel i underekstremiteten modsat den side, hvor transplantatet ventes indsat
3) Truende iskæmi i underekstremiteten

**Registrering.**
Ved inklusion registreres følgende oplysninger: alder, køn, højde, vægt, vævstypemismatch, vævstypeantistoffer (PRA), grundsygdom, restnyrefunktion, evt. dialysemodalitet (hæmodialyse/posedialyse), tidligere transplantationer, planlagt immunosuppresion, co-morbiditet (DM, hypertension, hypercholesterolæmi, aterosklerose i form af iskæmisk hjertesygdom, apoplexia cerebri eller perifer aterosklerose), status for infektion (serologi) med virus som CMV – dette med henblik på at vise at de to grupper er så ens som muligt.

Følgende oplysninger registreres vedrørende donor og donornyre: køn, alder, dødsårsag, p-kreatinin, vægt, varm og kold iskæmitid. Disse informationer registreres rutinemæssigt i forbindelse med transplantation, og der er således ikke tale om at indhente særskilte oplysninger om donor til nærværende studie.

**Kontakt til og forløb for forsøgsdeltager.**
Patienten kontaktes i forbindelse med akut indlæggelse til nekronyretransplantation. Den relevante mundtlige information om projektet samt udlevering af skriftligt materiale (deltagerinformation og "Forsøgspersonens rettigheder i et biomedicinsk forskningsprojekt" udgivet af Den Centrale Videnskabsetiske Komite) varetages af den speciallæge i urinvejskirurgi, som skal forestå transplantationen. I enkelte situationer vil informationen blive varetaget af projektansvarlige Nicoline V. Krogstrup eller en læge ansat på Nyremedicinsk afdeling C, dette kan forekomme fx ved stor travlhed hos kirurgen. Informationen foregår på sengestuen og så vidt muligt i enerum uden forstyrrende aktivitet på stuen. Patienten, som oftest medbringer pårørende til sygehuset, informeres om retten til bisidder. Patienten kan herefter afslå, give sit samtykke med det samme eller udbede sig betænkningstid indtil operationen. Såfremt patienten ønsker betænkningstid, vil denne blive adspurgt igen i forbindelse med klargøring til operationen under rolige omstændigheder på operationsgangen. Der vil samtidig være mulighed for at stille uddybende spørgsmål i forbindelse med projektet og patienten har herefter mulighed for at give sit samtykke eller afslå. Da operationen ofte udføres akut, er der ikke mulighed for længere betænkningstid.
Patienten informeres om, at 1) deltagelse i forsøget ikke nødvendigvis indebærer nogen fordel for patienten i forbindelse med transplantation 2) at deltagelse er frivillig og at dennes valg er uden indflydelse på dennes rettigheder og muligheder samt videre behandlingsforløb samt 3) at denne til enhver tid mundtligt, skriftligt eller ved anden klar tilkendegivelse, kan trække sit samtykke om at deltage tilbage.

Inkluderede patienter vil herefter blive randomiseret til enten rIC eller non-rIC.

Patienter, der randomiseres til rIC, vil få påsat en blodtomheds-manchet på benet (modsat den side, hvor nyren indsættes) som opblæses til 250 mmHg i 5 minutter efterfulgt af 5 minutters reperfusion. Dette gentages op til ialt 4 gange (3). Remote IC indledes når kirurgen påbegynder karanastomoseringen og afsluttes ideelt 10 minutter før reperfusion af nyren. Selve transplantationsoperationen, herunder perioden hvor nyren er udsat for kold og varm iskæmi, påvirkes i øvrigt ikke af forsøget.

Patienter der randomiseres til non-rIC vil også få påsat en blodtomheds-manchet på benet, men med en pean sat på slangen til manchetten under afdækningen, således at kirurgen ikke kender til randomiseringen.

**Indsamling af prøver**

**Under og umiddelbart efter operation:**
Veneblodprøver udtages fra CVK, der anlægges standard som led i operationen. Urinprøver tages fra blærekateter. Der tages vævsbiopsi fra graftnyren umiddelbart før reperfusion og igen 30 minutter efter reperfusion. Anlæggelse af blærekateter og biopsitagning efter reperfusion er vanlig procedure.

**Under postoperativ indlæggelse:**
Der tages rutinemæssigt standardblodprøver 2 gange dagligt, herunder måling af P-kreatinin og væsketal. Ekstra blodprøver tages til måling af biomarkører for akut nyreskade og oxidativt stress i plasma. Der gøres endvidere rutinemæssigt døgnurinopsamling de første 4 dage til kreatininclearance. I forbindelse hermed udtages ekstra urinprøver til bestemmelse af markører for akut nyreskade i urinen. På 5. dagen tages spoturin prøve.
GFR bestemmes på dag 5 +/- 1 ved $^{51}$Cr-EDTA plasma clearance, ved manglende nyrefunktion på dette tidspunkt aflyses undersøgelsen. På dag 1, 3 og 5 +/- 1 tages blodprøver til vurdering af immunreaktiviteten – herunder til isolering af immunaktive celler i blodet.
På dag 6 +/- 1 gøres endvidere ultralydsvejledt nyrebiopsi under lokalbedøvelse i overensstemmelse med vanlige retningslinier herfor. Ifald patienten ønsker at deltage i studiet men ikke ønsker denne biopsi foretaget kan den fravælges og patienten kan stadig inkluderes i studiet.
Patientens indlæggelsestid eller behandling i øvrigt vil ikke blive påvirket af at deltage i forsøget.

**Ambulant opfølgning:**
I forbindelse med almindelig ambulant opfølgning får patienten foretaget ny GFR måling ved $^{51}$Cr-EDTA clearance isotopteknik efter 3 måneder. GFR måling gentages igen 1 år efter transplantationen, dette vil ofte være en selvstændig besøgsdag. GFR måling kræver anlæggelse af drop. 1 måned, 3 måneder og et år efter transplantationen tages ekstra blodprøver som fryses. Der sendes urinprøve til rutineundersøgelse for albumin og en ekstra urinprøve sendes til frys. Det vil betyde et lidt længere ambulant besøg for patienten på de to dage end vanligt.

## ANALYSER OG BIOBANK

Som omtalt ovenfor tages en række blodprøver i forbindelse med nyretransplantation og efterforløbet som standardprocedure. Disse, herunder kreatinin i plasma og urin, vil indgå i forsøgets vurdering af nyrefunktionen. Supplerende blodprøver analyseres med henblik på måling af biomarkører (fx NGAL og 8-isoprostane), og bestemmelse af immunologiske markører, herunder isolation af myeloide og plasmacytoide DC i perifert blod med flowcytometrisk teknik. Ekstra blodprøver vil blive taget samtidig med standardblodprøver således at patienten ikke stikkes unødigt.
Nyrebiopsier taget under operationen samt på 6. dagen vil blive undersøgt af patolog efter vanlige retningslinier for vurdering af nyretransplant-biopsier. Biopsi på dag 6 vil gøre det muligt at vurdere, om rIC medfører strukturelle ændringer i nyren. Med henblik på at afsløre tegn på apoptose (celledød) og til at vurdere ekspressionen af relevante molekyler i relation til iskæmi-reperfusionsskader, herunder HIF, vil en del af biopsien blive præpareret til immunhistokemi ligesom en del af biopsien fryses ved -80 grader med henblik på senere ekspressionsanalyser ved qPCR.
Til opbevaring af blod- og urinprøver samt biopsier oprettes en biobank. Alle prøver vil blive opbevaret i en anonymiseret form frem til 2023, hvorefter de destrueres efter vanlige retningslinier for biologisk materiale. I fald forsøgsdeltageren ønsker at udtræde af forsøget, vil materialet ligeledes blive destrueret.

## RISICI VED FORSØGET

Blodtomhedsmanchet bruges i andre sammenhænge, f.eks. til ortopædkirurgiske operationer. Ved længerevarende brug over 1-2 timer med konstant blodtomhed kan ses lette hudskader hvis huden er blevet foldet under en manchet, der ikke er påsat korrekt. Der vurderes ikke at være nogen risiko for patienten ved rIC som beskrevet, da proceduren er kortvarig og afbrudt af fri blodtilførsel. Interventionen rIC er ikke forbundet med ubehag for patienten der er bedøvet under proceduren.

Ekstra nyregraftbiopsi taget før reperfusion er ligeledes forbundet med en lille risiko for blødning fra nyren. Da biopsien tages i god tid inden lukning af såret vil betydende blødning efter reperfusion af nyren kunne opdages og stoppes inden lukning af cicatricen. Der tages endvidere supplerende nyrebiopsi på dag 6 +/- 1. Denne tages under iagttagelse af sædvanlige forholdsregler, herunder forprøver, der skal sikre, at blodets evne til at koagulere er normal og dermed minimere risikoen for blødning. Der vil i forbindelse med biopsien være en lille risiko for blødning. Risikoen for en mindre blødning, typisk i form af forbigående hæmaturi udgør 1-2%, mens risikoen for større, transfusions- og evt. interventionskrævende blødning er < 0,1%. Biopsien foretages under lokalbedøvelse og er forbundet med mindre ubehag i form af smerter, samt besvær som følge af efterfølgende observationsperiode på 6 timer, hvor patienten skal være sengeliggende.
Blod- og urinprøver til forsøget tages så vidt muligt i forbindelse med rutineprøver og forventes ikke at være forbundet med yderligere risiko eller ubehag. De ekstra blodprøver udgør en volumen på max. 150 ml samlet, peroperativt og seks dage efter transplantation.
$^{51}$Cr-EDTA plasma clearance er en rutineundersøgelse og indebærer injektion af et radioaktivt lægemiddel. Strålingsdosis er ca. 0,01 mSv per undersøgelse – dette er mindre end ved en lille røntgenundersøgelse, og svarer til, hvad vi modtager fra naturen i løbet af 1 dag.
Indlæggelsesperioden vil ikke blive påvirket af deltagelsen i studiet.

## ETISKE ASPEKTER

Studiet forventes ikke at påvirke udkommet af transplantationsforløbet for forsøgsdeltagerne i negativ retning. Evt. positiv effekt for deltagerne vil afhænge af effekten af rIC. I forbindelse med forsøget følges patienterne mere intensivt end vanligt, herunder med $^{51}$Cr-EDTA plasma clearance og nyrebiopsi på 6. dagen. Resultatet af biopsien vil normalt ikke blive inddraget i den kliniske vurdering af patienten, med mindre der er problemer i forløbet, hvor den vil kunne bruges til gavn for patienten. Øvrige fund kan indgå i tilrettelæggelsen af patientens behandling, herunder justering af den immundæmpende behandling, og således potentielt kunne optimere forløbet for den enkelte patient.
På sigt vil forsøget forhåbentlig give mulighed for at forbedre transplantationsforløbet og mindske risikoen for nyresvigt efter transplantation med de fordele der er beskrevet nedenfor.

**Anmeldelse og godkendelse.** Projektet forudsætter godkendelse af den regionale Videnskabsetiske Komite og Datatilsynet. Enhver komplikation ved metoden vil blive vurderet af en speciallæge og vil løbende blive registreret og indberettet til Videnskabsetisk komite.
**Honorering for deltagelse.** Deltagere vil ikke blive honoreret for deres deltagelse i forsøget.
**Datatilsynet.** Oplysninger om patienten beskyttes efter lov om behandling af personoplysninger og sundhedsloven, og forsøget anmeldes til datatilsynet.

## PERSPEKTIVER

Forebyggelse af DGF og rejektion har vidtgående positive konsekvenser for både den enkelte patient og samfundet. Der forventes: 1) færre komplikationer (fx. infektioner og cancer som følge af mindre behov for immundæmpende behandling), 2) færre diagnostiske og terapeutiske interventioner, 3) mindre fysisk og psykisk belastning af patienterne i form af midlertidig dialyse, usikkerhed om forløb m.v., 4) længere funktionstid for transplanterede nyrer og 5) store både individuelle og samfundsmæssige økonomiske besparelser. Desuden vil forsøget kunne give en større forståelse for det tidlige immunologiske respons efter nyretransplantation, der på sigt kan bane vejen for længere og bedre funktion af transplanterede nyrer uden yderligere brug af medicin.

De potentielle fordele for fremtidige patienter og mulige samfundsøkonomiske gevinster synes således klart at opveje de risici og ulemper der er forbundet med forsøget.

**MULIGHED FOR GENNEMFØRELSE**
Der transplanteres i alt cirka 150 patienter årligt med nyre fra hjernedød donor på de to centre og det er planen at inkludere 200 patienter i forsøget. Kirurger er begge steder meget erfarne og indstillede på gennemførelse af projektet. Projektgruppen er del af et tværfagligt forskningsmiljø og der er et godt samarbejde mellem de involverede nyremedicinske, urologiske og anæstesiologiske afdelinger. Professor Bente Jespersen og overlæge Øyvind Østraat har i en årrække arbejdet med nyretransplantation. Henrik Birn og Rikke Nørregaard har lang forskningserfaring i basal nyrefysiologi og mekanismen bag renal tubulære funktion og skader. Nicoline Krogstrup har netop gennemført introduktionsstilling i nefrologi og er pt. ansat i et forskningsstipendium mhp. opgørelse af resultaterne vedrørende shocknyremarkører fra de nævnte grisenyretransplantationer. Hun vil kunne ansættes som klinisk assistent ved nyremedicinsk afdeling C i det påtænkte phd forløb.
GFR målinger vil blive udført af Nuklearmedicinsk afdeling, Skejby Sygehus. Klinisk Immunologisk afdeling, Skejby Sygehus, huser det nødvendige apparatur og ekspertise til flowcytometri udviklet i forbindelse med nævnte grisenyretransplantationsmodel.
Klinisk brug af prækonditionering er et fokusområde ved Århus Universitetshospital, hvor der også studeres effekter på hjerte og hjerne. Ph.d. studerende Kirsten Rønholt undersøger i øjeblikket, hvorvidt rIC kan forhindre nyrefunktionspåvirkning efter hjerteoperationer på børn, og i forbindelse med disse studier er markøren for renal iskæmi NGAL etableret i nefrologisk laboratorium.
Der er truffet aftale med klinikchef, professor Michael Olausson fra Transplantationsenheden, Sahlgrenska om inklusion af patienter fra Göteborg, og det aktuelle studium vil skabe mulighed for et tæt samarbejde både klinisk og forskningsmæssigt mellem de to centre. Desuden er der etableret samarbejde med professor Rutger Ploeg's gruppe i Groningen, Holland, med fokus på brug af biomarkører til prognosticering i forbindelse med nyretransplantation.

**PUBLIKATION**
Der forventes international publicering af forskningsresultaterne med projektansvarlig som førsteforfatter. Medforfattere inkluderes jfr. Vancouver reglementet. Resultaterne offentliggøres uanset om de er positive eller negative.

**ØKONOMI**
En stor del af udgifterne dækkes af de kliniske afdelinger i henholdsvis Skejby og Sahlgrenska idet mange af prøverne og undersøgelserne er en del af det almindelige, kliniske forløb ved nyretransplantation. Den projektansvarlige løn forventes dækket via Århus Universitetet i forbindelse med ansættelse som klinisk assistent. Forskningslaboratorium C (tilknyttet nyremedicinsk afd.) har et basisregnskab til drift af forskellige forskningsprojekter. Herudover søges forskellige fonde hvoraf Aarhus Universitet har doneret 80.000 kroner til etablering af det beskrevne samarbejde mellem Sahlgrenska og Aarhus Universitet. Lundbeckfonden har doneret 1.000.000 kroner og Novonordiskfonden 200.000 kroner til projektet.

**Litteraturreferencer**

1) Guimarães-Souza N, Dalboni MA, Canziani ME, Tedesco-Silva H, Batista MC, Sesso R, Câmara NO, Medina-Pestana JO, Pacheco-Silva A, Cendoroglo M. Clinical implications of initial renal function after deceased donor transplant.Transplant Proc. 2010 May;42(4):1084-9.
2) Loverre A, Capobianco C, Stallone G, Infante B, Schena A, Ditonno P, Palazzo S, Battaglia M, Crovace A, Castellano G, Ranieri E, Schena FP, Gesualdo L, Grandaliano G. Ischemia-reperfusion injury-induced abnormal dendritic cell traffic in the transplanted kidney with delayed graft function.Kidney Int. 2007;72(8):994-1003.
3) Bøtker HE, Kharbanda R, Schmidt MR, Bøttcher M, Kaltoft AK, Terkelsen CJ, Munk K, Andersen NH, Hansen TM, Trautner S, Lassen JF, Christiansen EH, Krusell LR, Kristensen SD, Thuesen L, Nielsen SS, Rehling M, Sørensen HT, Redington AN, Nielsen TT. Remote ischaemic conditioning before hospital admission, as a complement to angioplasty, and effect on myocardial salvage in patients with acute myocardial infarction: a randomised trial. Lancet. 2010 Feb 27;375(9716):727-34.
4) Kharbanda RK, Nielsen TT, Redington AN. Translation of remote ischaemic preconditioning into clinical practice. Lancet. 2009 Oct 31;374(9700):1557-65.
5) Cheung MM, Kharbanda RK, Konstantinov IE, Shimizu M, Frndova H, Li J, Holtby HM, Cox PN, Smallhorn JF, Van Arsdell GS, Redington AN. Randomized controlled trial of the effects of remote ischemic preconditioning on children undergoing cardiac surgery: first clinical application in humans. J Am Coll Cardiol. 2006 Jun 6;47(11):2277-82.
6) Shimizu M, Konstantinov IE, Kharbanda RK, Cheung MH, Redington AN. Effects of intermittent lower limb ischaemia on coronary blood flow and coronary resistance in pigs. Acta Physiol (Oxf). 2007 Jun;190(2):103-9.
7) Konstantinov IE, Li J, Cheung MM, Shimizu M, Stokoe J, Kharbanda RK, Redington AN. Remote ischemic preconditioning of the recipient reduces myocardial ischemiareperfusion injury of the denervated donor heart via a Katp channel-dependent mechanism. Transplantation 2005;79(12):1691-5.
8) Ali ZA, Callaghan CJ, Lim E, Ali AA, Nouraei SA, Akthar AM, et al. Remote ischemic preconditioning reduces myocardial and renal injury after elective abdominal aortic aneurysm repair: a randomized controlled trial. Circulation 2007 Sep 11;116(11 Suppl):I98-105.
9) Kanoria S, Jalan R, Seifalian AM, Williams R, Davidson BR. Protocols and mechanisms for remote ischemic preconditioning: a novel method for reducing ischemia reperfusion injury. Transplantation 2007 Aug 27;84(4):445-58.
10) Aydin Z, van Zonneveld AJ, de Fijter JW, Rabelink TJ. New horizons in prevention and treatment of ischaemic injury to kidney transplants. Nephrol.Dial.Transplant. 2007;22(2):342-6.
11) Resultaterne er netop præsenteret ved "XXV Scandinavian Transplantation Society Congress" i Helsinki d. 20. maj 2010 og ved "XXIII International Congress of The Transplantation Society", i Vancouver august 2010.
12) Iguchi M, Kakinuma Y, Kurabayashi A, Sato T, Shuin T, Hong S-B, Schmidt LS, Furihata FM. Acute Inactivation of the VHL Gene Contributes to Protective Effects of Ischemic Preconditioning in the Mouse Kidney. Nephron Exp Nephrol 2008;110:e82–e90
13) Bernhardt WM, Campean V, Kany S, Jurgensen JS, Weidemann A, Warnecke C, et al. Preconditional activation of hypoxia-inducible factors ameliorates ischemic acute renal failure. J Am Soc Nephrol 2006 Jul;17(7):1970-8.
14) Weidemann A, Bernhardt WM, Klanke B, Daniel C, Buchholz B, Câmpean V, Amann

K, Warnecke C, Wiesener MS, Eckardt KU, Willam C. HIF activation protects from acute kidney injury. J Am Soc Nephrol. 2008 Mar;19(3):486-94.
15) Jantsch J, Chakravortty D, Turza N, Prechtel AT, Buchholz B, Gerlach RG, Volke M, Gläsner J, Warnecke C, Wiesener MS, Eckardt KU, Steinkasserer A, Hensel M, Willam C. Hypoxia and hypoxia-inducible factor-1 alpha modulate lipopolysaccharide-induced dendritic cell activation and function. J Immunol. 2008 Apr 1;180(7):4697-705.
16) Schlichting CL, Schareck WD, and Weis M: Renal Ischemia–Reperfusion Injury: New Implications of Dendritic Cell–Endothelial Cell Interactions. Transplantation Proceedings 2006; 38:670-3.
17) Benichou G, Takizawa PA, Olson CA, McMillan M, Sercarz EE. Donor major histocompatibility complex (MHC) peptides are presented by recipient MHC molecules 18 of 23
during graft rejection. J Exp Med 1992;175:305–308
18) Soni SS, Cruz D, Bobek I, Chionh CY, Nalesso F, Lentini P de Cal M, Corradi V, Virzi G, Ronco C. NGAL: a biomarker of acute kidney injury and other systemic conditions. Int Urol Nephrol. 2010 Mar;42(1):141-50.
19) Che M, Xie B, Xue S, Dai H, Qian J, Ni Z, Axelsson J, Yan Y. Clinical usefulness of novel biomarkers for the detection of acute kidney injury following elective cardiac surgery. Nephron Clin Pract. 2010;115(1):c66-72.
20) Barklin A, Larsson A, Vestergaard C, Koefoed-Nielsen J, Bach A, Nyboe R, Wogensen L, Tonnesen E. Does brain death induce a pro-inflammatory response at the organ level in a porcine model? Acta Anaesthesiol.Scand. 2008;52(5):621-7.
21) Keller AK, Jorgensen TM, Ravlo K, Nielsen TK, Olsen LH, Stolle LB. Microdialysis for detection of postoperative ischemia in experimental renal transplantation. J Urol. 2009 Oct;182(4 Suppl):1854-9.

## Lægmandsresumé

Projektets formål er at forbedre og forlænge funktionen af transplanterede nyrer uden yderligere brug af medicin, og at klarlægge vigtige tidlige reaktioner i immunsystemet efter en nyretransplantation.
Kronisk nyresvigt er forbundet med højere dødelighed end de fleste cancersygdomme. Den bedste behandling af kronisk nyresvigt er nyretransplantation, alternativet er dialysebehandling som er meget belastende for den enkelte patient og omkostningstung (ca. 400.000 kr. årligt pr. patient). Nyretransplantation er forbundet med langt lavere omkostninger, forbedret prognose og markant bedre livskvalitet. Nedsat funktion og afstødning giver imidlertid fortsat store problemer, så den transplanterede nyre typisk kun fungerer i 10 år.
Hvis nyren har forsinket igangsætning, som det ses hos 25% af organmodtagerne fra hjernedød donor, øges risikoen for afstødning. Denne dvaletilstand af nyren, hvor den ikke danner urin de første dage-uger efter transplantationen kaldes shocknyre. Shocknyre skyldes iltmangel i nyrevævet og skader i forbindelse med den første gennemblødning af nyren i modtageren.
Det er netop blevet påvist af vores samarbejdspartnere i Skejby, at ”remote ischemic conditioning” (rIC) som består af gentagne afklemninger af blodforsyningen til en arm kan beskytte hjertet mod skader ved blodprop i hjertet. Vi har etableret en grisemodel for nyretransplantation fra hjernedød donor, hvor vi har kunnet påvise, at rIC på modtageren øgede tidlig nyrefunktion.
Vi ønsker nu at inkludere 200 patienter, der transplanteres med nyre fra en afdød donor, i et studie. Halvdelen af organmodtagerne får efter lodtrækning rIC i benet på den side nyren ikke indsættes. rIC er uden bivirkninger og er ikke til gene for patienten da det udføres under operation mens patienten er bedøvet. Under vanlig indlæggelse efter en transplantation får patienterne målt nyrefunktionen og forskellige markører for immunsystemet samt shocknyremarkører.
De ekstra blodprøver forbundet med forsøget udgør for patienten en ubetydelig volumen. Analysering af de forskellige markører vil ikke få nogen betydning for patientens øvrige sygdomsforløb. Præcis måling af nyrefunktionen foregår med en sporstofmetode ($^{51}$Cr-EDTA plasma clearance) der medfører minimal stråledosis. Stråledosis per undersøgelse (3 i alt) er mindre end ved en lille røntgenundersøgelse, og svarer til, hvad vi modtager fra naturen i løbet af 1 dag. Der tages biopsi fra nyren under operationen hvilket er standardprocedure, samt igen seks dage efter. Biopsien på dag 6 tages ultralydsvejledt efter brug af lokalbedøvelse. Der kan være lidt ubehag forbundet med biopsitagningen og der er en lille risiko for blødning, men biopsi fra den transplanterede nyre er rutine og tages ofte i forvejen en uge efter transplantation for at udelukke afstødning. Det er muligt at fravælge biopsien på 6. dagen.
Efterfølgende måles nyrefunktionen igen efter tre måneder og efter et år i forbindelse med ambulant kontrol, hvilket kræver anlæggelse af drop i hånden. Der tages også ekstra blod- og urinprøver. Ifald der er problemer i efterforløbet af transplantationen vil den ekstra biopsi taget i forbindelse med studiet på 6. dagen kunne bruges som led i den kliniske vurdering til gavn for patienten.

Vi forventer at studiet vil vise at rIC, der er en enkel procedure at udføre og som er gratis og uden bivirkninger, vil kunne bedre funktionen af transplanterede nyrer og at de heraf affødte fordele klart opvejer eventuelle risici.
Projektet søges finansieret gennem fondsstøtte fra offentlige eller private, almennyttige fonde, således har Lundbeckfonden doneret 1 mio. kroner og Novonordiskfonden 200.000 kroner. Der betales ingen vederlæg hverken til patienter eller til læger involveret i patienternes behandling.
